# *Syzygium cumini* (L.) skeels: a prominent source of bioactive molecules against cardiometabolic diseases

**DOI:** 10.3389/fphar.2015.00259

**Published:** 2015-11-03

**Authors:** Vinicyus Teles Chagas, Lucas Martins França, Sonia Malik, Antonio Marcus de Andrade Paes

**Affiliations:** ^1^Laboratory of Experimental Physiology, Department of Physiological Sciences, Federal University of MaranhãoSão Luís, Brazil; ^2^Graduate Program in Health Sciences, Biological and Health Sciences Center, Federal University of MaranhãoSão Luís, Brazil

**Keywords:** black plum, jamun, myrtaceae, phenolic compounds, metabolic syndrome, ethnopharmacology, medicinal plants, complementary and alternative medicine

## Abstract

*Syzygium cumini* (Myrtaceae) is a worldwide medicinal plant traditionally used in herbal medicines due to its vaunted properties against cardiometabolic disorders, which include: antihyperglycemic, hypolipemiant, antiinflammatory, cardioprotective, and antioxidant activities. These properties have been attributed to the presence of bioactive compounds such as phenols, flavonoids, and tannins in different parts of the plant, albeit the knowledge on their mechanisms of action is scarce. This mini-review highlights the cardiometabolic properties of *S. cumini* by correlating its already identified phytochemicals with their described mechanisms of action. Data herein compiled show that some compounds target multiple metabolic pathways; thereby, becoming potential pharmacological tools. Moreover, the lack of clinical trials on *S. cumini* usage makes it a fruitful field of interest for both scientific community and pharmaceutical industry.

## Introduction

Cardiometabolic syndrome is associated with multiple risk factors including insulin resistance, dyslipidemia, hypertension, and obesity (Alberti et al., 2009). According to World Health Organization, every year about 2.8 million people die worldwide due to overweight or obesity (Lim et al., 2012). Prevalence of diabetes appears with projections to affect about 439 million adults by 2030, whereas cardiovascular diseases account for 30% of deaths annually, including both developed and developing countries (Shaw et al., 2010). Because of their chronic degenerative nature, cardiometabolic-related disorders have long-lasting treatments, costly for both the patient and the health services, in addition to potentially harmful side effects caused by polytherapeutic regimens (Yanovski and Yanovski, 2014). In this context, herbal medicines have become the major source of bioactive molecules and emerged as potential therapeutic tools to fulfill a multiple-target strategy, especially because of their inherented large-scale structural diversity as compared with synthetic compounds (Prabhakar and Doble, 2008; Vasudeva et al., 2012).

Myrtaceae family comprises about 121 genera with 3800–5800 species of shrubs and trees distributed mainly in tropical and subtropical areas of the world (Stefanello et al., 2011). The genus *Syzygium*, a leading member of this family, embrace 1100 species with deserving attention to *Syzygium cumini* (L.) Skeels (syn.: *Eugenia jambolana, Syzygium jambolanum*), which has been used in the treatment of numerous diseases, especially diabetes (Ayyanar and Subash-Babu, 2012). The use of *S. cumini* was introduced in western medicine in the mid-nineteenth century, when the first reports on the investigation of its antidiabetic properties were published (Helmstadter, 2008). *S. cumini* is a large tree native from Indian subcontinent, but widely cultivated in many countries in Asia, Africa, and South America (Srivastava and Chandra, 2013). It is popularly known as jamun in India, black plum in Europe, jambolan in Spanish-spoken countries and jambolão in Brazil (Corrêa, 1974).

*S. cumini* is known to possess wide range of medicinal properties, which have been attributed to the presence of bioactive compounds in different parts of the plant. The leaves are used in dermopathies, gastropathies, constipation, leucorrhea, and diabetes; fruits are used in the treatment of pharyngitis and splenic diseases; whereas barks are used as astringents, anthelmintic, and carminative. Furthermore, seeds are used as astringents, diuretic, and especially in the treatment of diabetes (Warrier, 1997; Helmstadter, 2008; Baliga et al., 2011). Pharmacological studies have expanded the biological activities of *S. cumini*, which include antihyperglycemic, antiinflammatory, antibacterial, cardioprotective, and antioxidant (Kumar et al., 2008a; Rekha et al., 2008; Sharma et al., 2008b, 2011; Mastan et al., 2009; Arun et al., 2011; Tanwar et al., 2011). Likewise, some remarkable and well-conducted literature surveys on pharmacological properties, phytochemical constituents as well as nutritive value of *S. cumini* have been published along the last decade (Helmstadter, 2008; Ayyanar and Subash-Babu, 2012; Baliga et al., 2013; Srivastava and Chandra, 2013).

Notwithstanding those findings, however, there is scarcity of data on the relationship of its secondary metabolites with the described biological effects, especially on the mechanisms of action of these compounds. Therefore, this mini-review is aimed to specifically study the cardiometabolic properties of secondary metabolites already identified in this plant species, correlating them with their potential mechanisms of action. For this purpose, a detailed literature survey has been carried out using both preclinical and clinical studies as an attempt to identify the molecular mechanisms of action for the compounds identified in the various parts of *S. cumini*, even considering they may not have been necessarily isolated from this plant species, but had the same chemical identity of those found in it.

## Phytochemical Constituents

Various secondary metabolites viz., flavonoids, phenolic acids, tannins, and terpenes have been reported in different parts of *S. cumini* (**Table 1**). For instance, leaves of this plant species contain high levels of flavonoids, especially quercetin, myricetin, myricitrin, kaempferol, and their glucoside derivatives, in addition to simple phenols like ellagic acid, ferulic acid, chlorogenic acid, and gallic acid (Mahmoud et al., 2001; Timbola et al., 2002; Ruan et al., 2008). The essential oil of leaves is prevalent in terpenes such as α-pinene, β-pinene, α-limonene, α-cadinol, pinocarvone, pinocarveol (Shafi et al., 2002; Mohamed et al., 2013). The seeds are the most studied part of the plant, being especially high in hexahydroxydiphenic (HDDP) acid-derivated hydrolyzable tannins, terpenes like as α-terpineol, eugenol, betulinic acid, and the same abovementioned phenolic acids (Bhatia and Bajaj, 1975; Ramya et al., 2012). Flowers have been found to show a very similar chemical composition to seeds, although pharmacological and chemical studies on this part are scarce (Baliga et al., 2011; Gordon et al., 2011). In addition, fruits also contain anthocynins, like as cyanidin, delphinidin, and petudinine, which give them a bright violet color (Ramya et al., 2012). Stem bark has essentially the same phenolic acids, flavonoids, and terpenes described already for other parts of *S. cumini* (Bhatia and Bajaj, 1975; Baliga et al., 2011).

**Table 1:** Phytochemical compounds identified in different parts of *Syzygium cumini*.

Plant part	Metabolite class	Identified compounds	Reference
Leaf	Flavonoids	Catechin, kaempferol, myricetin, myricetin 3-O-β-D-glucuronopyranoside, myricetin-4′-methyl ether 3-*O*-α-rhamnopyranoside, myricetrin 4″-*O*-acetate, myricetrin 4″-*O*-acetyl-2-*O*-gallate, myricitrin, quercetin -3-*O*-α-rhamno_ pyranoside	Mahmoud et al., 2001
	Phenolic acids	Caffeic acid, chlorogenic acid, ellagic acid, ferulic acid, gallic acid	Mahmoud et al., 2001; Timbola et al., 2002; Ruan et al., 2008
	Tannins	Nilocetin	Mahmoud et al., 2001
	Terpenes	α-pinene, α-cadinol, pinocarvone, pinocarveol, α-terpeneol, myrtenol, eucarvone, muurolol, myrtenal, cineole, geranylacetone	Shafi et al., 2002; Mohamed et al., 2013
Seed	Flavonoids	Quercetin, rutin, 3,5,7,4-tetrahydroxy flavanone	Bhatia and Bajaj, 1975; Karthic et al., 2008
	Phenolic acids	Caffeic acid, ellagic acid, ferulic acid, gallic acid	Bhatia and Bajaj, 1975
	Tannins	Corilagin, 3,6-HHDP glucose, 4,6-HHDP glucose, 1-galloyl glucose, 3-galloyl glucose	Bhatia and Bajaj, 1975
	Terpenes	α-terpineol, β-pinene, β-terpinene, betulinic acid, eugenol	Williamson et al., 2002; Karthic et al., 2008
Fruit	Flavonoids	Myricetin, myricetin deoxyhexoside	Gordon et al., 2011
	Phenolic acids	Ellagic acid, gallic acid	Reynertson et al., 2008; Gordon et al., 2011
	Tannins	HHDP-galloyl glucose, trigalloylglucose	Gordon et al., 2011
	Terpenes	Citronellol, geraniol, hotrienol, nerol, β-phenylethanol, phenylpropanal	Vernin et al., 1991
	Anthocyanins	Cyanidin, delfinidin, petudinin	Veigas et al., 2007
Flower	Flavonoids	Kaempferol, myricetin, dihydromyricetin, myricetin-3-L-arabinoside, isoquercetin, quercetin, quercetin-3-D-galactoside	Subramanian and Nair, 1972
	Phenolic acids	Ellagic acid	Baliga et al., 2011
	Terpenes	Eugenol, oleanolic acid	Ramya et al., 2012
Stem bark	Flavonoids	Myricetin, quercetin, kaempferol	Baliga et al., 2011
	Phenolic acids	3,3′-di-*O*-methyl ellagic acid, 3,3′, 4-tri-*O*-methyl ellagic acid, gallic acid	Bhatia and Bajaj, 1975; Ramya et al., 2012
	Terpenes	β-siterol, friedelin, betulinic acid	Ramya et al., 2012

*HHDP, hexahydroxydiphenic acid.*

## Cardiometabolic Properties and Potential Mechanisms of Action

### Antihyperglycemic Activity

**FIGURE 1:**
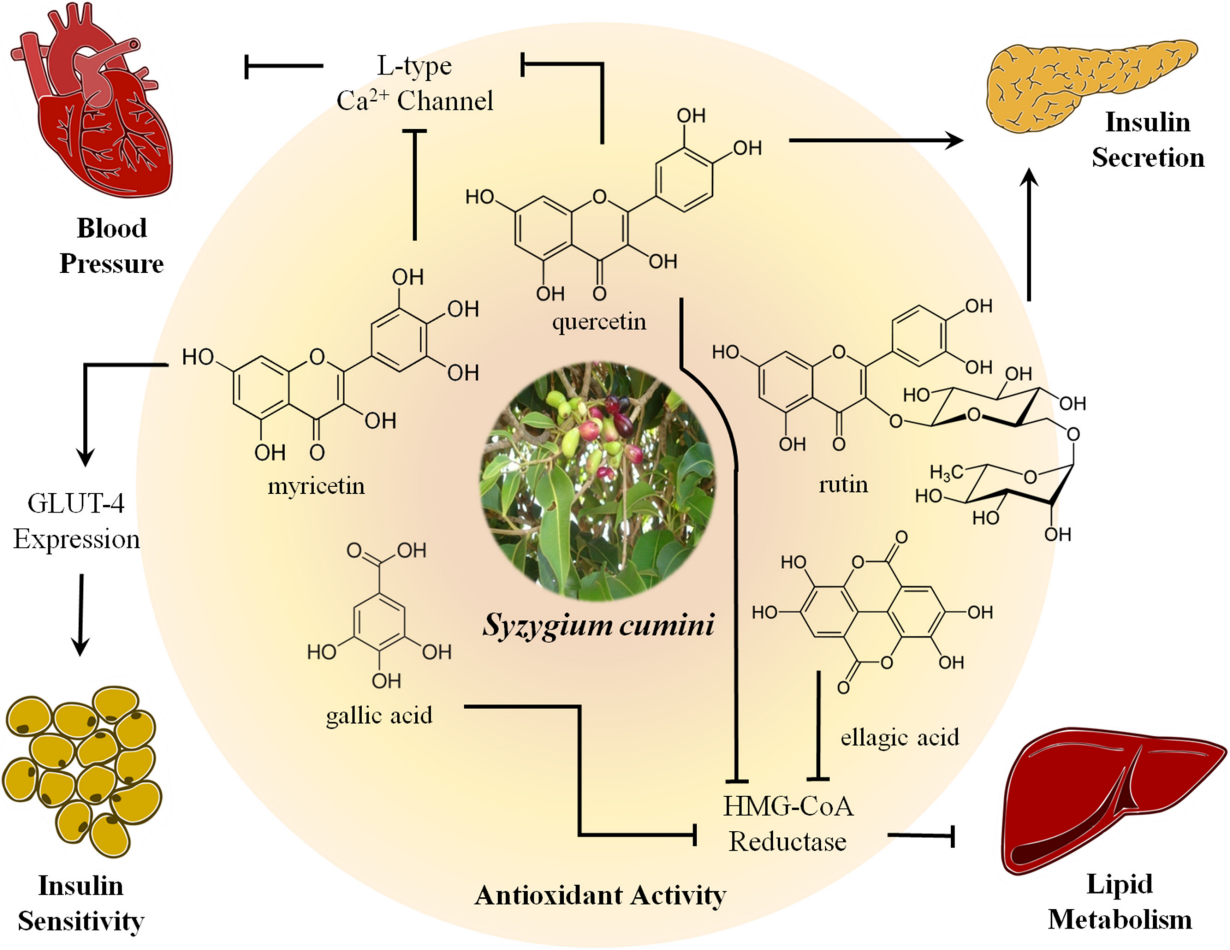
**Biological properties and main mechanisms of action described for the most prevalent polyphenolic compounds identified in *Syzygium cumini*.** Polyphenolic compounds identified in different parts of *S. cumini* are shown and connected to target mechanisms and/or organs by arrowheadlines for activating pathways and interrupted lines for inhibiting pathways. Antihyperglycemic effects of quercetin and rutin have been ascribed to the stimulus of insulin secretion through blockage of L-type calcium channels on pancreatic beta cells, a mechamism also shared by quercetin and myricetin for vasorelaxant and antihypertensive properties. Quercetin and phenolic acids, such as ellagic and gallic acids, inhibit 3-hydroxy-3-methyl-glutaryl (HMG)-CoA reductase in the liver, concurring for antihyperlipidemic properties. Myricetin also improves glucose homeostasis by stimulation of GLUT-4 expression in both adipose tissue and skeletal muscle. Notwithstanding the abovementioned effects, all the showed compounds possess well-characterized antioxidant activity, which is also expected to underlie these cardiometabolic properties of *S. cumini*.

Use of *S. cumini* in the fight against diabetes has been studied by western medicine since more than 130 years (Helmstadter, 2008). In recent years, numerous preclinical studies have evaluated extracts of various parts, especially seeds, of this plant species for anti-hyperglycemic activity (Ravi et al., 2003; Schossler et al., 2004; Anandharajan et al., 2006; Sharma et al., 2008a,b; Ramya et al., 2012; Silva et al., 2012). Blood and urine glucose levels of streptozotocin-induced diabetic rats were decreased upon 30-days treatment with ethanolic extract of seed at doses of 100 mg/kg/day (Ravi et al., 2003). In addition to blood glucose lowering effect, flavonoid-rich extract of seed was also shown to recover peripheral glucose tolerance in streptozotocin-induced diabetic rats (500 mg/kg/day, 21 days) (Sharma et al., 2008a) and mice (300 mg/kg/day, 15 days) (Sharma et al., 2008b). Those effects were ascribed to increased activity of peroxisome proliferator-activated receptors (PPAR) alpha and gamma, which was assessed in 3T3-L1 preadipocytes incubated for 24 h with increasing concentrations (1–100 mg/mL) of flavonoid-rich extract of seed. In this same study, seed extract was shown to possess insulinotropic activity, which may be involved in the abovementioned effects (Sharma et al., 2008a). Considering flavonoids already identified in *S. cumini* seed (**Table 1**), antihyperglycemic effect of rutin was attributed to inhibition of glucose metabolism enzymes hexokinase and glucose-6-phosphatase (Kamalakkannan and Prince, 2006). As highlighted in **Figure 1**, rutin (0.5–8 mM) also increased insulin secretion in streptozotocin-treated pancreatic islets of rats (Esmaeili et al., 2009). On the other hand, 20 μM quercetin stimulated insulin secretion by activation of L-type calcium channels (**Figure 1**) in isolated rat beta cells (Bardy et al., 2013). Nevertheless, quercetin has also been described to increase insulin sensitivity by improving the production of the adipocyte-derived factors, like adiponectin and leptin (Wein et al., 2010).

*S. cumini* has also been described to promote other insulin-related effects. Incubation of methanolic extract of leaf (1 ng/mL-10 μg/mL) with cultured L6 myotubes for 0.5 and 24 h was found to increase mRNA expression of glucose transporter (GLUT)-4 and phosphatidylinositol 3-kinase (PI3 kinase), both important mediators of insulin action in adipocytes and skeletal muscle (Anandharajan et al., 2006). Myricetin (1 mg/kg, 3× per day, 14 days), one of the most prevalent flavonoids in the leaf, has been shown to improve GLUT-4 expression in both adipose tissue and skeletal muscle as well (**Figure 1**; Liu et al., 2007). Flavonoid-rich extract of *S. cumini* leaf (300 mg/kg/day, 15 days) reduced by 50% the expression of aldose reductase in renal tissue of diabetic rats (Sharma et al., 2008b), an effect previously described for phenolic compounds, such as myricetin, quercetin, kampferol, and ellagic acid (Haraguchi et al., 1998). The use of *S. cumini* (300 mg/kg/day, 15 days) elevated the concentrations of glycogen in liver and skeletal muscle, suggesting a stimulatory effect on glycogen synthase or glycogenolysis inhibition (Sharma et al., 2008b). These effects have been described for oleanolic acid (5–100 μM) (Ha et al., 2009) and caffeic acid (200 g/kg/day, 5 weeks) (Jung et al., 2006), compounds found in flowers and seeds, respectively. *In vitro* studies showed that betulinic acid and 3,5,7,4′-tetrahydroxy flavanone isolated from *S. cumini* seed inhibited pancreatic α-amylase, which may cause diminished intestinal absorption of carbohydrates (Karthic et al., 2008). Nevertheless, bark extract (1 g/kg/day, 30 days) was shown to regenerate cells from pancreatic duct (Schossler et al., 2004).

### Antihyperlipidemic Activity

Flavonoid-rich extract of *S. cumini* seed (300 mg/kg/day, 15 days) was described to reduce total cholesterol, LDL-cholesterol, and triacylglycerol as well as raise HDL-cholesterol levels (Sharma et al., 2008b). Similar results were found for aqueous extract of fruit at doses of 100 and 200 mg/kg (Rekha et al., 2008), for hydroalcoholic extract of seed kernel (100 mg/kg/day, 30 days) (Ravi et al., 2005) and for administration of Dihar (100 mg/kg/day, 6 weeks), an Indian mixture of herbs containing *S. cumini* (Patel et al., 2009). In all the abovementioned studies, dyslipidemia secondary to streptozotocin-induced diabetes was used as animal model to assess *S. cumini* antihyperlipidemic activity, which have been mainly ascribed to the inhibition of 3-hydroxy-3-methyl-glutaryl-CoA (HMG-CoA) reductase (**Figure 1**), the enzyme responsible for cholesterol synthesis (Ravi et al., 2005; Sharma et al., 2008b, 2011). Flavonoids found in *S. cumini* (**Table 1**) are expected to account for this activity, since it has been described that this class of compounds increases the expression of cAMP-dependent phosphokinase, enzyme responsible for HMG-CoA reductase inhibition (Havsteen, 2002). However, such effects might also be due reduction of intestinal absorption of cholesterol, as well as, increased free fatty acid and triacylglycerol clearances subsequent to insulin action improvement. (Ravi et al., 2005; Birari and Bhutani, 2007; Sharma et al., 2012). In fact, preliminary data from our group has pointed out that hydroethanolic extract of *S. cumini* leaf inhibits both activity and expression of hepatic microsomal triglyceride-transfer protein, which is further controlled by insulin signaling pathways. Nevertheless, quercetin has been recently shown to prevent OP9 mouse stromal cells differentiation into adipocytes by downregulation of adipogenic genes (Seo et al., 2015).

### Cardioprotective Activity

*S. cumini* has also been reported to promote hypotensive and antihypertensive effects. Chronic administration of hydroalcoholic extract of *S. cumini* leaf (100 and 250 mg/kg/day, 20 weeks) reduced blood pressure in normotensive rats, an effect further corroborated by decreased reactivity of vascular smooth muscle observed upon incubation of its chloroform (0.25 and 0.5 mg/mL) and aqueous fractions (0.1, 0.25, and 0.5 mg/mL; Ribeiro, 2007). More recently, the same group showed a dose-dependent reduction of blood pressure and heart rate on spontaneously hypertensive rats submitted to 8-weeks treatment with the same extract (0.5 g/kg/day) (Ribeiro et al., 2014). In both studies, authors speculated the extract might contain some compound able to non-competitively blockade L-type calcium channel. As depicted in **Figure 1**, myricetin has been reported to promote potent vasodilation by impairment of calcium influx (Herrera et al., 1996), similarly to quercetin, whose vasospasmolytic effect has been ascribed to blockage of calcium influx through L-type calcium channels (Hou et al., 2014).

Besides its effects on vasomotricity, *S. cumini* seems to improve hemodynamics as well. In a recent work, platelets collected from diabetic patients were incubated with aqueous extract of *S. cumini* leaf (100 and 200 μg/mL) resulting in lower platelet aggregation and decreased oxidative damage, as assessed by measurement of lipoperoxide and nitric oxide levels and superoxide dismutase activity. The extract was still found to increase platelet cell membrane fluidity and to stimulate Na^+^/K^+^-ATPase activity (Raffaelli et al., 2014). In earlier work, De Bona et al. (2010) had already reported that same concentrations of aqueous extract of *S. cumini* leaf decreased adenosine deaminase and 5′-nucleotidase activities, important thrombogenic enzymes, when incubated with platelets from diabetic patients. Phenolic compounds identified in the extract, such as gallic acid, chlorogenic acid, and rutin, were suggested as possible candidates for the abovementioned activities. Moreover, administration of methanolic extract of seed (200 mg/kg) previously to isoproterenol-induced infaction in rats, reduced serum levels of myocardial necrosis biomarkers, specifically aspartate aminotransferase, alanine aminotransferase, uric acid, creatine phosphokinase, and lactate dehydrogenase (Mastan et al., 2009). Notwithstanding, administration of α-hydroxy cinnamic acid (10, 15, and 20 mg/kg/day, 30 days), isolated from aqueous extract of *S. cumini* fruit, to high fat-fed diabetic rats promoted antiatherosclerotic effects mainly characterized by decreased oxidized LDL levels, modulation of endothelial nitric oxide synthase, lower expression of soluble vascular cell adhesion molecule-1 and significant reduction of atherogenic lipoprotein apolipoprotein B_100_ along with an increase of apolipoprotein A_1_ (Tanwar et al., 2011).

### Antiinflammatory Activity

Inflammatory processes are directly involved in the development of cardiometabolic diseases including atherosclerosis, type 2 diabetes, and cancer (Hotamisligil, 2006). Methanolic and ethyl acetate extracts of *S. cumini* leaf (200 and 400 mg/kg) reduced carrageenan-induced paw edema in rats (Jain et al., 2011). Methanolic extract of seed (250 and 500 mg/kg, 21 days) also reduced paw edema volume and leukocyte migration in rats with adjuvant-induced arthritis (Kumar et al., 2008b). Ethanolic extract of the bark (100, 300, and 1000 mg/kg) reduced the production of prostaglandin E2, serotonin, and histamine (Muruganandan et al., 2002). Similarly, pre-treatment of mice with ethanolic extract of leaf (25, 50, and 100 mg/kg) reduced systemic production of pro-inflammatory factors, such as interleukin-5 (Brito et al., 2007), whereas TNF-α and NO^⋅^ production was decreased at doses of 5 or 50 mg/kg administered 6-h previous to inflammation induction (Maciel et al., 2008).

### Antioxidant Activity

Polyphenolic and related antioxidant compounds are recognized as important cardiometabolic agents since they scavenge reactive oxygen/nitrogen species and stimulate antioxidant defenses (Valko et al., 2007), which may be involved in all the aforementioned activities of *S. cumini* (**Figure 1**). Oral administration of aqueous extract of seed (500, 1000, and 1500 mg/kg/day, 5 days) to mice treated with urethane 7,12-dimethyl benzanthracene resulted in reduced chromosomal damage, significantly inhibited hepatic lipid peroxidation, which was associated with significantly increased activity of glutathione *S*-transferase, superoxide dismutase, and catalase (Arun et al., 2011). Pre-treatment of cyclophosphamide-administered rats with methanolic extract of *S. cumini* fruit (100 and 200 mg/kg/day, 14 days) resulted in reduced formation of hepatic malondialdehyde, lower frequency of aberrant metaphases, reduced formation of micronuclei, decreased cytotoxicity against marrow cells and increased activity of antioxidant enzymes, like as superoxide dismutase and catalase, besides increased GSH levels (Tripathi et al., 2013).

Considering *in vitro* studies, methanolic extract of *S. cumini* leaf and branch (1–15 μg/ml) has been shown to strongly act against OH^⋅^ and DPPH^⋅^ radicals, and decreased Fe^3+^ to Fe^+2^ reduction in the FRAP assay. Such activities showed a strong correlation with the high content of polyphenols and flavonoids present in the extract (Eshwarappa et al., 2014). In another report, antioxidant activity of ethyl acetate fraction (15.6–125 μg/mL) prepared from methanolic extract of *S. cumini* leaf was also correlated with its polyphenolic composition, especially to ferulic acid and catechins present in the extract (Ruan et al., 2008). Aqil et al. (2012) reported that ethanolic extracts of fruit and seed, at different concentrations, showed strong antioxidant activity assessed by mean of distinct assays, such as ABTS^⋅+^, DPPH^⋅^, FRAP, and ORAC. These extracts also showed antiproliferative activity on lung cancer cells, making them potential sources of anticarcinogenic agents. Anthocyanins (delphinidin, cyanidin, petunidin, peonidin, and malvidin), besides ellagic acid and its derivatives (ellagitannins) identified in both extracts were suggested to be responsible for the antioxidant and antiproliferative activities (Aqil et al., 2012). Antioxidant activity of *S. cumini* fruit has also been shown to be due to gallic acid, as well as, derivatives of tetragalloyl glucose and myricetin (Gordon et al., 2011).

## Toxicity

Several studies have shown that *S. cumini* do not produce acute or chronic toxicity when given by oral route. General toxicological screenings including behavioral, histomorphological as well as blood hematological and biochemical parameters have been conducted for extracts from seed (Chaturvedi et al., 2007), fruit (Kumar et al., 2008a), and leaf (Silva et al., 2012) of *S. cumini*, with no toxic effect noticed. Silva et al. (2012) demonstrated that acute administration of hydroalcoholic extract of leaf at doses as high as 2 g/kg produced no toxic effects in rodents. The same study demonstrated that chronic treatment for up to 180 days at doses of 50, 100, and 250 mg/kg did not cause hematological, biochemical, or histological alterations in target organs (Silva et al., 2012). These results are further corroborated by findings of potential hepatoprotective effect of ethanolic extract of *S. cumini* fruit, without any harmful side effects in animals (Bilal et al., 2011).

## Clinical Studies

Notwithstanding all the accumulated knowledge on the pharmacological properties of *S. cumini*, very limited data has come from clinical trials (Helmstadter, 2008). A study conducted by Srivastava et al. (1983) showed that acute administration of capsuled seed powder (4–24 g) to patients with severe diabetes notably reduced blood glucose levels in both fasting and post-prandial condictions. Similarly, Kohli and Singh (1993) reinforced the effects of seed powder (12 g/day, 3 months) on type 2 diabetes mellitus patients by showing that besides a 30% reduction of serum glucose levels, treatment also improved other classical symptoms of diabetes, like polyphagia, polyuria, and polidisia. On the other hand, a single administration of decocted dried powdered leaves of *S. cumini* (2 g in 250 mL water) to young normoglycemic patients had no effect on serum glucose levels (Teixeira et al., 2000). In a second approach, Teixeira et al. (2006) administered the same decoction to type 2 diabetic patients for 28 days observing no effect on glucose levels. Albeit contradictory results showed above, the plethora of preclinical studies on the cardiometabolic properties of *S. cumini* supports the necessity of well-designed trials that allow an efficient assessment of its therapeutic potentials in humans. In addition, standardization of extraction method and characterization of phytochemicals present in the extracts are fundamental to the success of such trials.

## Conclusion

The present work enlightens cardiometabolic properties described for *S. cumini*, which have been attributed to a limited amount of phytochemicals, particularly flavonoids, phenolic acids, and tannins (**Table 1**). As summarized in **Figure 1**, some compounds like myricetin, quercetin, rutin, ellagic, and gallic acids seem to be able to act on distinct pathways of cardiometabolic disorders, thus emerging as potential multi-targeted drugs. Nevertheless, the knowledge on their precise mechanisms of action is scanty and still deserves in-depth scientific research, especially concerning their jointed action as a phytocomplex. Finally, though toxicological studies have shown the species safety, clinical trials are barely inexistent pointing out a golden Eldorado for pharmaceutical companies.

## Conflict of Interest Statement

The authors declare that the research was conducted in the absence of any commercial or financial relationships that could be construed as a potential conflict of interest.
